# Assessment of Open-Angle Glaucoma Peripapillary and Macular Choroidal Thickness Using Swept-Source Optical Coherence Tomography (SS-OCT)

**DOI:** 10.1371/journal.pone.0157333

**Published:** 2016-06-16

**Authors:** Yong Ju Song, Young Kook Kim, Jin Wook Jeoung, Ki Ho Park

**Affiliations:** Department of Ophthalmology, Seoul National University College of Medicine, Seoul, Korea; Charité University Medicine Berlin, GERMANY

## Abstract

**Objective:**

To compare peripapillary and macular choroidal thickness (PCT and MCT) between open-angle glaucoma (OAG) and normal controls using swept-source optical coherence tomography (SS-OCT), and to evaluate global and localized relationships between choroidal thickness and various factors in OAG, also using SS-OCT.

**Methods:**

In this cross-sectional comparative study, 134 OAG patients and 73 normal controls were examined. PCT (global, 12 clock-hour sectors), MCT (global, six sectors) were measured by SS-OCT. The difference in choroidal thickness between the OAG patients and the normal controls was analyzed. The relationships between choroidal thickness and various factors including age, sex, spherical equivalent (SE), axial length (AXL), central corneal thickness (CCT), intraocular pressure (IOP), peripapillary retinal nerve fiber layer thickness (pRNFLT), visual field mean deviation (MD), ganglion cell-inner plexiform layer thickness (GCIPLT), and disc area were analyzed by univariate and multivariate linear regression. Global and regional analyses were performed in 12 segments of the peripapillary circle and in six sectors of the macula.

**Results:**

There were significant differences in global PCT and MCT between the OAG patients and the normal controls (115.22±41.17 vs. 138.89±44.70, P<0.001), (184.36±57.15 vs. 209.25±61.11, P = 0.004). The difference in global PCT remained, both after adjusting for age, AXL (117.08±3.45 vs. 135.47±4.70, P = 0.002) and also after adjusting for age, AXL, disc area (117.46±3.46 vs. 135.67±4.67, P = 0.002). But the difference in global MCT did not remain after adjusting for age, AXL, SE (188.18±4.46 vs. 202.25±6.08, P = 0.066). PCT showed significant differences between the groups in all of the 12 clock-hour sectors. These differences remained after adjusting for age, AXL and for age, AXL, disc area, with the exception of the 10 o’clock (o/c) sector. MCT in six sectors showed differences between the two groups, but they did not remain after adjusting for age, AXL, SE. In a multivariate regression analysis of the OAG patients, global PCT showed correlations with age (β = -1.18, P = 0.001), AXL (β = -14.01, P<0.001), and disc area (β = -16.67, P = 0.026). Global MCT, meanwhile, showed a significant correlation with age (β = -1.92, P<0.001), AXL. (β = -21.97, P<0.001). Choroidal thickness did not show any global or localized relationship with glaucoma severity in the OAG patients.

**Conclusions:**

The global and all 12 clock-hour PCT, with the exception of the 10 o/c sector, were thinner in OAG; however, they did not show any correlation with glaucoma severity. Possible roles of PCT in glaucoma pathogenesis should be investigated further.

## Introduction

Glaucoma is a leading cause of blindness worldwide, and its pathophysiology is still not completely clear.[1] Glaucoma is a progressive optic neuropathy related to retinal ganglion cell (RGC) death in the optic nerve head (ONH) with loss of visual field.[2] As elevated intraocular pressure (IOP) is a main risk factor for progression of glaucoma (and a main influence, therefore, on prognosis), its control is a mainstay of glaucoma treatment.[2] Despite adequate IOP control (within the low teens) though, glaucoma progression sometimes occurs. In line with this, many studies have focused on ocular ischemia and the role of ocular blood supply in glaucomatous optic nerve damage.[3–5] The choroid is the abundant vascular layer, the blood flow of which is the highest per unit weight in the human body. The choroid, connecting the ora serrata to the optic disk, provides for more than 70% of the eye’s circulatory blood. In this way, it supplies nutrients to the outer retina and ONH, especially the prelaminar region, which is closely related to RGC death in glaucoma.[6, 7]

Several techniques have been employed for choroid evaluation in cases of glaucoma: histology, [8] radiofrequency measurement,[9] Doppler flowmetry,[3] and optical coherence tomography (OCT). Yin et al. demonstrated choroidal thinning with concomitant reduction of choroidal vessel diameter and density in glaucoma patients.[8] Cristini et al. reported 20% increase of choroidal thickness in glaucoma patients compared with normal controls.[9] Based on a flowmetry study comparing glaucoma patients with normal controls, Grunwald et al. reported diminished blood flow to the ONH in glaucoma patients, but without any difference in choroidal flow to the fovea.[3] Among them, OCT emerged as the most useful method for measurement of choroidal thickness in vivo, thanks to OCT-technological improvements such as enhanced depth imaging (EDI) and swept-source OCT (SS-OCT). Most OCT studies have found that there is no difference in peripapillary or macular choroidal thickness (PCT, MCT) between open-angle glaucoma (OAG) patients and normal controls, and also that there is no relationship between choroidal thickness and glaucoma severity.[10–18] But some studies have reported thinning of PCT or MCT in OAG patients,[19–22] and Hirooka et al. reported a relationship between choroidal thickness and glaucoma severity especially in the nasal region 3 mm from the fovea, which is close to the peripapillary choroid, which fact might affect the ONH blood supply.[23]

In the previous OCT studies, the relationship between choroidal thickness and glaucoma was evaluated according to choroidal thickness parameters and visual field mean deviation (MD). However, as glaucomatous change usually is focal at the early-to-moderate stage, the results of those previous studies cannot be considered to represent any exact relationship between choroidal thickness and glaucoma. In the present study, we employed the parameters, PCT and peripapillary retinal nerve fiber thickness (pRNFLT), as measured in the 12 clock-hour sectors of the peripapillary circle, as well as the parameters, MCT and ganglion cell-inner plexiform layer thickness (GCIPLT), as measured in the six sectors of the macula. Additionally, in those same sectors, we analyzed the relationships between choroidal thickness and various factors.

## Methods

### Subjects

This was a cross-sectional comparative study. One hundred and thirty-four (134) eyes of 134 OAG patients and 73 eyes of 73 normal controls who had visited Seoul National University Hospital between October 2013 and February 2015 were included. The study was conducted in compliance with the tenets of the Declaration of Helsinki, and was approved by the Institutional Review Board of Seoul National University Hospital, Korea. Written informed consent was obtained from all subjects.

All subjects underwent a comprehensive ophthalmologic examination including best-corrected visual acuity (BCVA), refractive error with an autorefractor (KR-890; Topcon, Inc., Tokyo, Japan), IOP measurement by Goldmann applanation tonometry, gonioscopy, dilated fundus examination and standard automated perimetry (SAP) using the Swedish Interactive Threshold Algorithm 30–2 (Humphrey Field Analyzer; Carl Zeiss Meditiec, Inc., Dublin, CA), as well as central corneal thickness (CCT) with pachymetry (POCKET II pachymeter echograph; Quantel Medical, Inc., Clermont-Ferrand, France), and axial length (AXL) measurement with ocular biometry (IOL Master; Carl Zeiss Meditiec Inc., Dublin, CA). PCT, MCT, pRNFLT, GCIPLT were measured using swept-source Deep Range Imaging OCT (DRI-OCT-1 Atlantis; Topcon, Inc., Tokyo, Japan).

The following individuals were selected according to the following inclusion criteria: (1) open angle on gonioscopic examination, (2) BCVA ≥20/40, (3) SE within ± 6.50 diopters and cylinder correction within ± 3.00 diopters, (4) AXL ≤ 26.5 mm, (5) SS OCT image quality ≥40. The exclusion criteria were ocular surgery history, closed angle chamber, and any ocular or systemic disease possibly affecting the retina or optic nerve.

The selected glaucoma patients all showed representative glaucomatous optic disc change (neuroretinal rim thinning, notching, or RNFL defect) and accompanying visual field defect, as detected repeatedly by reliable SAP measurements in consecutive visual fields. If both eyes were glaucomatous, one eye was randomly selected as the study eye. The normal controls was defined as those with IOP ≤ 21 mmHg with no history of elevated IOP, no visible RNFL defect on red-free fundus photography, and a normal SAP result.

### Swept-source OCT scanning protocols and segmentation procedure

Retinal and choroidal images were acquired by SS-OCT, which utilizes a wavelength swept laser as a light source, with a 1050 nm center wavelength for easy visualization of deeper structures including the choroid. SS-OCT also provides a fast scanning speed of 100,000 A-scans/sec, an axial resolution of 8 um, and a wide, 3–12 mm scan range horizontally and vertically.

In this study, 3-dimensional (3D) optic disc- and wide-scanning protocols were applied. First, the 3D optic disc scan, entailing a 6x6 mm raster scan centered on the optic disc, delivered 3-dimensional (3D) images composed of 256 B-scans, each B-scan comprising 512 A-scans, for a total of 131,072 axial scans/volume. Second, the wide scan employed was a 12x9 mm raster scan centered on the posterior pole. This mode provided 3D images of the same composition as in the first mode.

SS-OCT segmentation software (version 9.11, Topcon, Inc., Tokyo, Japan) was used to identify the limits of the choroid and to determine choroidal thickness. The data was exported using the manufacturer’s OCT-Batch (version 9.1.10) utility. The quality of each scan and the accuracy of the segmentation algorithm were determined by masked reviewers (YJS and KHP). Poor-quality (image quality <40), segmentation-failure images were excluded from further analysis.

**Fig 1:**
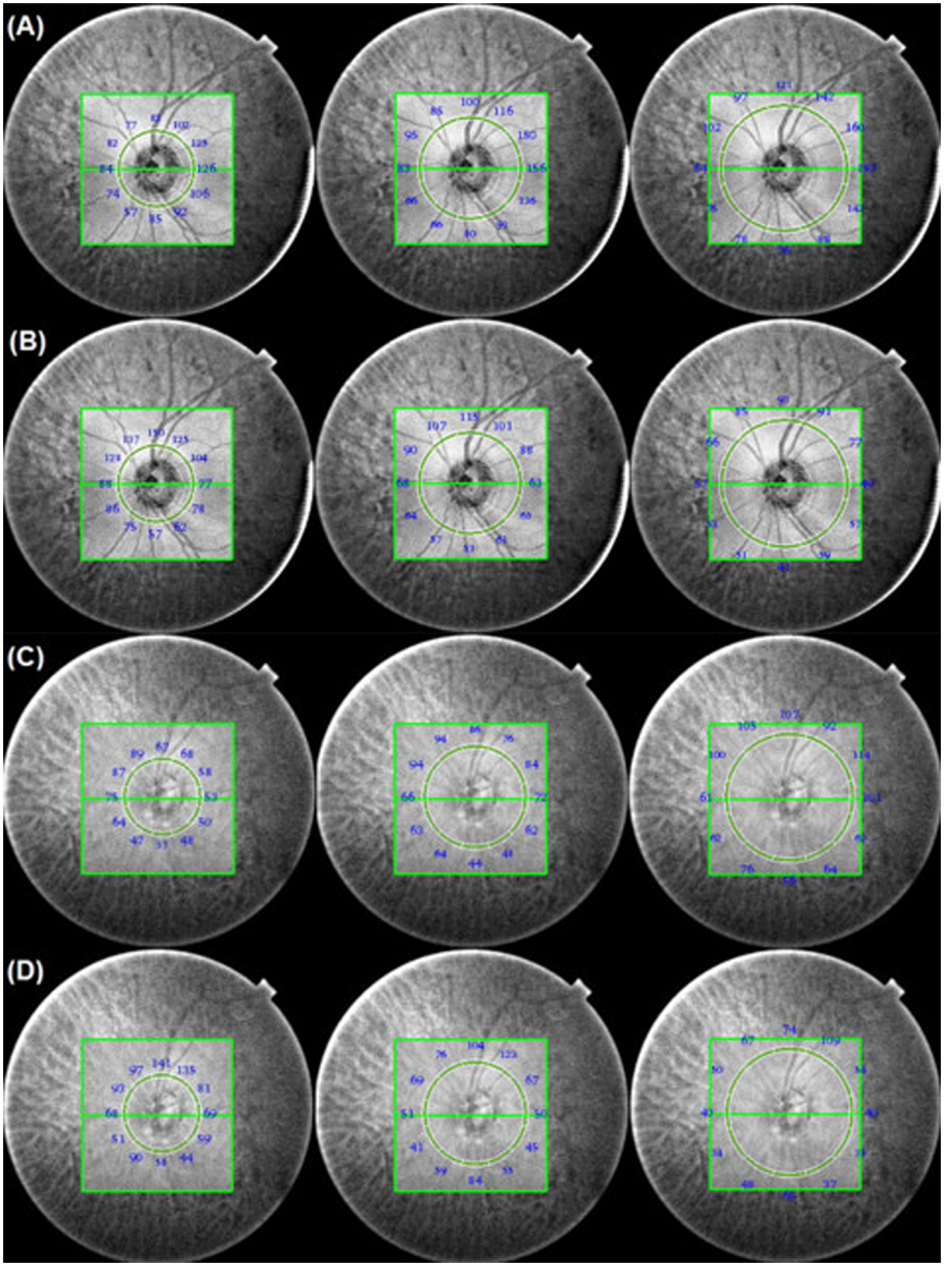
Measurement of peripapillary choroidal thickness (PCT) and retinal nerve fiber layer thickness (RNFLT) at diameters of 3, 4, 5 mm using 6x6 mm Optic disc scan. (A) 62-year-old normal control with axial length of 23.42 mm. The global PCT was 101.72 um. (B) The global RNFLT of the normal control was 79.94 um. (C) 61-year-old normal-tension glaucoma (NTG) patient with visual field defect (mean deviation, -5.98 dB) and axial length of 23.25 mm. The global PCT was 72.11 um (D) The global RNFLT of the NTG patient was 68.50 um.

After segmentation, all 12 clock-hour pRNFLT, PCT were calculated automatically by means of 3, 4, 5 mm diameter peripapillary circles centered on the optic disc (Fig 1). The localized pRNFLT, PCT were defined as the mean thickness when measured in increasing increments of 3, 4 and 5 mm as performed in a clock-wise fashion from the center of the circle in each clock-hour sector. The global pRNFLT, PCT were estimated as the mean thicknesses of the localized pRNFLT, PCT.

For both the right and left eyes, 12 o’clock (o/c) corresponded to the superior region, 3 o/c to the nasal region, 6 o/c to the inferior region, and 9 o/c to the temporal region. The right-eye orientation was used in documenting all of the OCT data.

**Fig 2:**
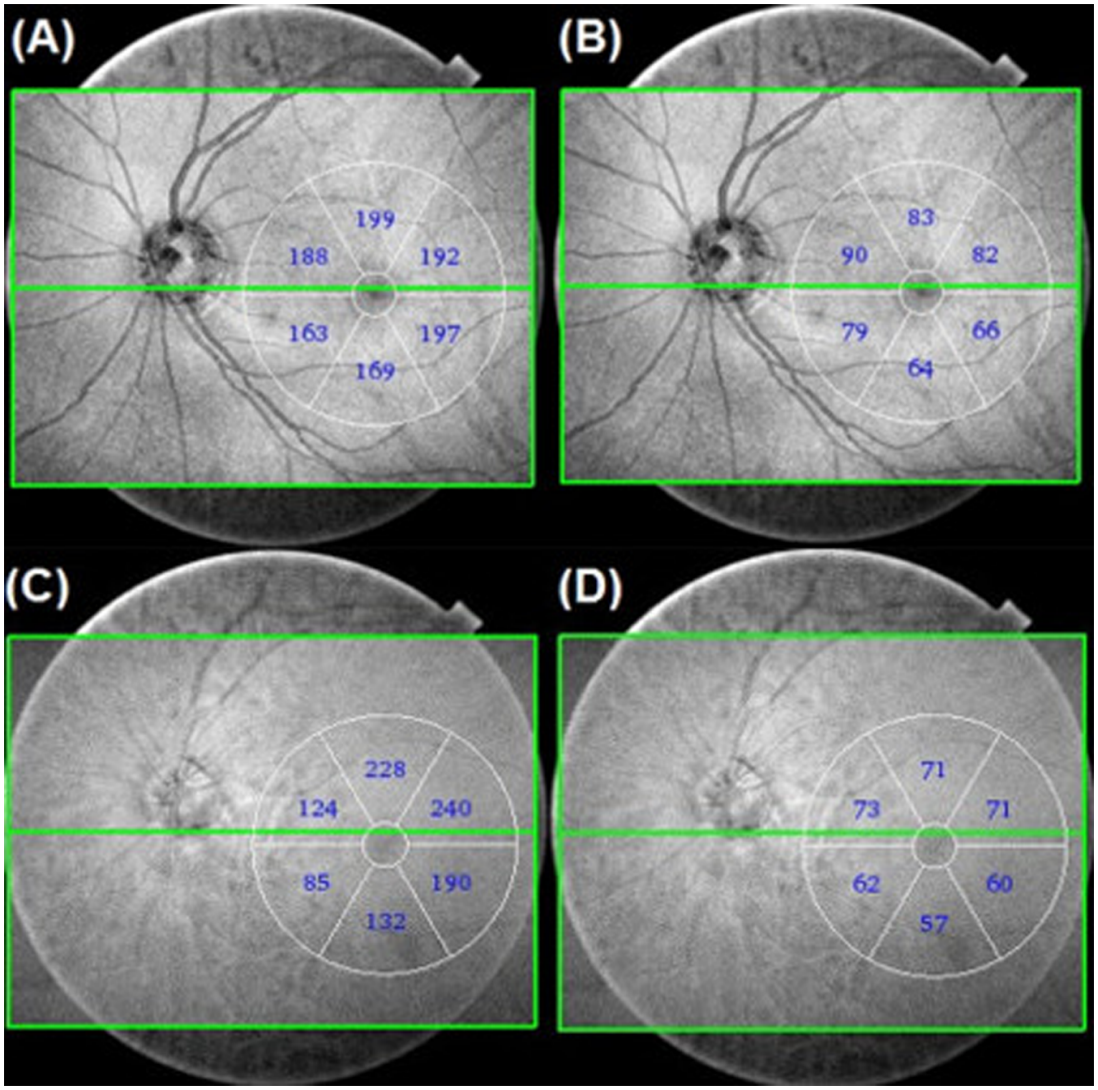
Measurement of macular choroidal thickness (MCT) and ganglion cell-inner plexiform layer thickness (GCIPLT) within 6x6 mm diameter parafoveal circle using 12x9 mm Wide scan. (A) 62-year-old normal control with axial length of 23.42 mm. The global MCT was 184.66 um. (B) The global GCIPLT of the normal control was 77.33 um. (C) 61-year-old NTG patient with visual field defect (mean deviation, -5.98 dB) and axial length of 23.25 mm. The global MCT was 166.50 um. (D) The global GCIPLT of the NTG patient was 65.67 um.

In the six (6) sectors (superotemporal, superior, superonasal, inferotemporal, inferior, inferotemporal) of the MCT, GCIPLT was calculated automatically by way of a 6x6 mm diameter parafoveal circle centered on the fovea (Fig 2).

### Statistical analysis

The statistical analyses were performed using SPSS 19.0 (SPSS, Inc., Chicago, IL, USA). To compare the clinical characteristics between the glaucoma and normal control groups, the Student t-test was used for continuous variables, and the Pearson chi-square test for categorical variables. To compare the glaucoma and control groups for adjusted choroidal thickness, analysis of covariance was utilized. The relationships between choroidal thickness and various factors including age, SE, AXL, CCT, IOP, RNFLT, visual field MD, GCIPLT, and disc area were analyzed by univariate and multivariate linear regression in both groups (glaucoma, normal control group). Variables showing a value of P<0.2 in the univariate regression were included in the subsequent multivariate regression. Both the univariate and multivariate regression analyses were performed according to the following OCT parameters: global choroidal thickness and localized thickness in the 12 clock-hour sectors of the peripapillary circle and the six sectors of the macula. In all of the analyses, values of P<0.05 were considered statistically significant.

## Results

### Demographic data on subjects

A total of 134 eyes of 134 OAG patients (114 NTG patients and 20 primary open-angle glaucoma (POAG) patients) and 73 eyes of 73 normal controls were included in this study. All of the subjects were Korean. The mean age of the OAG patients was 55.97 ± 11.41 years, and that of the normal controls was 55.56 ± 10.35 years. There were no significant differences in, age, sex, eye laterality, SE, CCT, IOP or disc area between the OAG patients and the normal controls. However, AXL, pRNFLT, GCIPLT, and MD showed significant differences between the two groups. The demographic data are summarized in Table 1.

**Table 1:** Demographics and ocular characteristics of subjects.

	OAG (n = 134) Mean ± Standard Deviation	Controls (n = 73) Mean ± Standard Deviation	P Value
**Age (years)**	55.97 ± 11.41	55.56 ± 10.35	0.800*
**Sex, female**	63 (47%)	35 (49%)	0.771†
**Eye laterality, right eye**	73 (55%)	35 (48%)	0.386†
**Spherical equivalent (Diopters)**	-2.54 ± 2.85	-1.76 ± 2.80	0.062*
**Axial length (mm)**	24.63 ± 1.17	24.25 ± 1.25	0.031*
**Mean CCT (μm)**	529.08 ± 33.95	537.49 ± 33.05	0.087*
**Mean IOP (mmHg)**	12.62 ± 2.47	12.53 ± 2.09	0.786*
**RNFL thickness (μm)**	69.97 ± 15.39	88.49 ± 11.76	<0.001*
**GCIPL thickness (μm)**	64.02 ± 7.13	70.64 ± 7.13	<0.001*
**MD (dB)**	-5.76 ± 5.22	-0.04 ± 2.14	<0.001*
**Disc area (mm**^**2**^**)**	1.90 ± 0.46	1.95 ± 0.39	0.445*

CCT = central corneal thickness; IOP = intraocular pressure; RNFL = retinal nerve fiber layer; GCIPL = ganglion cell-inner plexiform layer; MD = mean deviation; PCT = peripapillary choroidal thickness; MCT = macular choroidal thickness.

* Comparison of the 2 groups by Student-t test

^†^ Comparison of the 2 groups by Pearson chi-square test

### Choroidal thickness compared between OAG and normal controls

Between the glaucoma patients and normal controls, there was a significant difference in global PCT (115.22±41.17 vs. 138.89±44.70, P<0.001), as well as in MCT (184.36±57.15 vs. 209.25±61.11, P = 0.004). The difference in global PCT remained, both after adjusting for age, AXL (117.08±3.45 vs. 135.47±4.70, P = 0.002) and also after adjusting for age, AXL, disc area (117.46±3.46 vs. 135.67±4.67, P = 0.002). But the difference in global MCT did not remain after adjusting for age, AXL, SE (188.18±4.46 vs. 202.25±6.08, P = 0.066). PCT showed significant differences between the groups in all of the 12 clock-hour sectors. These differences remained after adjusting for age, AXL and for age, AXL, disc area, with the exception of the 10 o/c sector. MCT in six sectors showed differences between the two groups, but they did not remain after adjusting for age, AXL, SE.

In both groups, PCT was most thin in the inferior area and most thick in the superior area. Meanwhile, MCT was thinnest in the nasal area and thickest in the superior area. The choroidal thickness data comparison between the two groups is summarized in Tables 2 and 3.

**Table 2:** Choroidal global and adjusted thicknesses in OAG and normal controls.

	OAG (n = 134) Mean ± Standard Deviation	Controls (n = 73) Mean ± Standard Deviation	P Value
**PCT (μm)**	115.22 ± 41.17	138.89 ± 44.70	<0.001^†^
**Adjusted PCT (μm)**	117.08 ± 3.45*	135.47 ± 4.70*	0.002^‡^
**Adjusted PCT (μm)**	117.46 ± 3.46*	135.67 ± 4.67*	0.002^#^
**MCT (μm)**	184.36 ± 57.15	209.25 ± 61.11	0.004^†^
**Adjusted MCT (μm)**	188.18 ± 4.46*	202.25 ± 6.08*	0.066^$^

OAG = open-angle glaucoma; PCT = peripapillary choroidal thickness; MCT = macular choroidal thickness

* Expressed as mean ± standard error.

^†^ Comparison of the 2 groups by Student–t test

^‡^ Comparison of the 2 groups by Covariance (age, axial length) analysis test

^#^ Comparison of the 2 groups by Covariance (age, axial length, disc area) analysis test

^$^ Comparison of the 2 groups by Covariance (age, axial length, spherical equivalent) analysis test

**Table 3:** Choroidal localized and adjusted thickness in OAG and normal controls.

Peripapillary choroidal thickness (μm)						Macular choroidal thickness (μm)				
Location	OAG (n = 134)	Controls(n = 73)	P	P	P	Location	OAG (n = 134)	Controls (n = 73)	P	P
**12 o/c**	133.99 ± 48.58	157.60 ±57.96	0.002*	0.013^†^	0.008^‡^	**S**	201.17 ± 62.96	227.75 ± 66.34	0.005*	0.052^#^
**1 o/c**	133.47 ± 48.28	156.75 ± 58.38	0.002*	0.011^†^	0.008^‡^	**SN**	163.20 ± 61.22	191.37 ± 66.87	0.003*	0.053^#^
**2 o/c**	131.22 ± 48.36	154.98 ± 56.04	0.002*	0.006^†^	0.005^‡^	**IN**	155.31 ± 64.27	184.67 ± 69.41	0.003*	0.053^#^
**3 o/c**	121.81 ± 43.09	152.04 ± 52.19	<0.001*	<0.001^†^	<0.001^‡^	**I**	187.59 ± 64.83	210.91 ± 70.56	0.017*	0.165^#^
**4 o/c**	113.26 ± 45.15	138.84 ± 51.19	<0.001*	0.001^†^	0.001^‡^	**IT**	196.06 ± 65.08	215.50 ± 65.12	0.041*	0.289^#^
**5 o/c**	98.17 ± 41.84	120.31 ± 47.19	0.001*	0.003^†^	0.004^‡^	**ST**	202.85 ± 62.71	224.98 ± 65.60	0.018*	0.145^#^
**6 o/c**	85.21 ± 38.39	102.06 ± 38.54	0.003*	0.014^†^	0.018^‡^					
**7 o/c**	91.11 ± 45.14	110.39 ± 41.87	0.003*	0.025^†^	0.033^‡^					
**8 o/c**	103.34 ± 52.67	130.20 ± 51.56	0.001*	0.008^†^	0.012^‡^					
**9 o/c**	114.44 ± 51.00	142.72 ± 57.36	<0.001*	0.006^†^	0.009^‡^					
**10 o/c**	125.67 ± 55.42	148.30 ± 55.09	0.005*	0.057^†^	0.072^‡^					
**11 o/c**	130.70 ± 50.93	152.52 ± 52.84	0.004*	0.036^†^	0.036^‡^					

OAG = open-angle glaucoma; β = Beta; P = P value

* Comparison of the 2 groups by Student–t test

^†^ Comparison of the 2 groups by Covariance (age, axial length) analysis test

^‡^ Comparison of the 2 groups by Covariance (age, axial length, disc area) analysis test

^#^ Comparison of the 2 groups by Covariance (age, axial length, spherical equivalent) analysis test

### Relationship between choroidal thickness and various factors

In the univariate regression, the PCT of the OAG group showed a significant negative relation with AXL (Beta = -6.35, P = 0.036), while the PCT of the normal controls show a significant relation with age (Beta = -1.01, P = 0.045). In the multivariate regression, the PCT of the OAG group showed a significant relation with age (Beta = -1.18, P = 0.001), AXL (Beta = -14.01, P<0.001), and disc area (Beta = -16.67, P = 0.026). The PCT of the normal controls also showed a significant relation with age (Beta = -1.57, P = 0.003) and AXL (Beta = -12.32, P = 0.005).

In the univariate regression, the MCT of the OAG group showed a significant association with SE (Beta = 3.85, P = 0.026), AXL (Beta = -12.45, P = 0.003). The MCT of the normal controls also showed a relation with age (Beta = -1.51, P = 0.028), SE (Beta = 7.36, P = 0.003) and AXL (Beta = -16.93, P = 0.003). In the multivariate regression, the MCT of the OAG group had a relation with age (Beta = -1.92, P<0.001), AXL (Beta = -21.98, P<0.001). The MCT of the normal controls also had a relation with age (Beta = -2.67, P<0.001), AXL (Beta = -25.23, P<0.001).

In the multivariate regression analysis of all 12 clock-hour sectors of the peripapillary circle, thinner PCT in the OAG group was associated with older age in the 1, 2, 3, 4 and 12 o/c sectors, with longer AXL in the 7, 8, 9, 10, and 11 o/c sectors, and with large disc (disc area) in the 1, 2, 11, and 12 o/c sectors. Thinner MCT in the OAG group was associated with older age in the S, I, IT, and ST sectors, and with longer AXL in every sector. The data on the relationships between choroidal thickness and the various factors are summarized in Tables 4 and 5.

**Table 4:** Univariate regression analysis of choroidal thickness with associated factors.

	Peripapillary choroidal thickness				Macular choroidal thickness			
	OAG (n = 134)		Controls (n = 73)		OAG (n = 134)		Controls (n = 73)	
**Factors**	**β**	**P**	**β**	**P**	**β**	**P**	**β**	**P**
**Age (years)**	-0.57	0.064*	-1.01	0.045*	-0.78	0.073*	-1.51	0.028*
**SE (Diopters)**	2.03	0.104*	3.12	0.096*	3.85	0.026*	7.36	0.003*
**AXL (mm)**	-6.35	0.036*	-7.41	0.077*	-12.45	0.003*	-16.93	0.003*
**Mean CCT (μm)**	-0.03	0.741*	-0.14	0.383*	0.05	0.726*	0.13	0.544*
**Mean IOP (mmHg)**	0.67	0.644*	1.82	0.471*	0.70	0.729*	2.78	0.422*
**MD (dB)**	1.04	0.126*	0.95	0.701*	1.15	0.225*	0.579	0.864*
**RNFLT average (μm)**	0.06	0.777*	0.16	0.721*				
**Disc area (mm**^**2**^**)**	-12.29	0.112*	6.65	0.619*				
**GCIPL thickness (μm)**					1.51	0.082*	1.46	0.070*

OAG = open-angle glaucoma; SE = spherical equivalent; AXL = axial length; CCT = central corneal thickness; IOP = intraocular pressure; MD = mean deviation; RNFLT = retinal nerve fiber layer thickness; GCIPL = ganglion cell-inner plexiform layer; β = Beta; P = P value

* Analysis by univariate regression test

**Table 5:** Multivariate regression analysis of choroidal thickness with associated factors in each area.

Open-angle glaucoma (n = 134)							
Peripapillary choroidal thickness				Macular choroidal thickness			
Location	Factors	β	P	Location	Factors	β	P
**Global**	**Age**	-1.18	0.001*	**Global**	**Age**	-1.92	<0.001*
	**AXL**	-14.01	<0.001*		**AXL**	-21.98	<0.001*
	**Disc area**	-16.67	0.026*	**S**	**Age**	-1.91	<0.001*
**12 o/c**	**Age**	-0.73	0.043*		**AXL**	-16.03	0.002*
	**Disc area**	-24.81	0.006*	**SN**	**AXL**	-16.92	<0.001*
**1 o/c**	**Age**	-0.92	0.010*	**IN**	**AXL**	-19.24	<0.001*
	**Disc area**	-23.34	0.008*	**I**	**Age**	-1.86	0.001*
**2 o/c**	**Age**	-0.96	0.008*		**AXL**	-21.93	<0.001*
	**Disc area**	-19.31	0.030*	**IT**	**Age**	-2.30	<0.001*
**3 o/c**	**Age**	-0.96	0.003*		**AXL**	-23.31	<0.001*
**4 o/c**	**Age**	-0.82	0.017*	**ST**	**Age**	-2.46	<0.001*
**5 o/c**					**AXL**	-19.61	<0.001*
**6 o/c**							
**7 o/c**	**AXL**	-10.08	0.002*				
**8 o/c**	**AXL**	-14.14	<0.001*				
**9 o/c**	**AXL**	-13.50	<0.001*				
**10 o/c**	**AXL**	-12.91	0.001*				
**11 o/c**	**AXL**	-11.69	0.002*				
	**Disc area**	-26.08	0.007*				

AXL = axial length; S = superior; SN = superonasal; IN = inferonasal; I = inferior; IT = inferotemporal; ST = superotemporal; β = Beta; P = P value

* Analysis by multivariate regression test

## Discussion

In the present study, we found global PCT thinning in OAG patients relative to normal controls. And in the localized PCT analysis, all 12 clock-hour sectors of the PCT were thinner, except for the 10 o/c sector. The global, localized PCT in OAG patients were correlated with age, AXL, disc area but not with glaucoma severity (RNFLT or MD). These global, localized PCT thinning results are consistent with those of Park et al.,[21] who compared the PCT of 48 normal controls with those of NTG (n = 56) and POAG (n = 52) patients using Heidelberg Spectralis EDI-OCT. They measured PCT from the end adjacent to the ONH to a 3 mm distance from the center of the ONH at six points in four sectors (temporal, nasal, superior, inferior). They reported significant average PCT thinning in each of the four sectors in the NTG patients compared with the normal controls. However, there was no significant PCT difference between the POAG patients and the normal controls. In their mixed model, average PCT was correlated with age, AXL, glaucoma type but not with glaucoma severity (MD). We believe that the relative consistency of outcomes between Park et al. and the present study was due to the similar subject groups and peripapillary measurement adjacent to the ONH. Regarding the subject groups, the present study considered mostly NTG eyes (114 of 134 eyes). And as for the peripapillary measurement adjacent to the ONH, we measured the PCT, including a 1.5 mm distance from the disc center, located close to the disc margin. With respect to the ONH blood supply, Hayreh et al. suggested that branches of the short posterior ciliary artery and the circle of Zinn-Haller enter the peripapillary choroid, which in turn supplies blood to the ONH.[24] Thus, we considered that PCT measurement adjacent to the ONH can be a useful means of reflecting the ONH blood supply. As for the differences between the present study and Park et al.’s, first, Park’s study measured PCT manually, and present study by automated segmentation; second, Park et al.’s study did not investigate the relation between localized PCT and variable factors in NTG patients, which rendered determination of the localized relationship between choroidal thickness and glaucoma difficult. Although average PCT shows no correlation with global glaucoma severity, it is important to investigate whether PCT thinning is localized thinning corresponding to RNFL defect or a global change regardless of RNFL defect. In our present results, all 12 clock-hour sectors of PCT showed no localized RNFL thickness, indicating that PCT thinning was not a localized reduction but rather a global reduction regardless of RNFLD location. Hirooka et al.[19] also investigated the relationship between localized PCT thinning and glaucoma severity in NTG patients. They compared the PCT results for 50 normal controls with those for NTG (n = 52) patients using Heidelberg Spectralis EDI-OCT. Having measured PCT manually in eight sectors using a 360°, 3.4 mm diameter circle scan centered on the optic disc, they reported PCT thinning in the inferior sectors (inferonasal, inferior, inferotemporal) in NTG patients compared with normal controls. They noted also that the total deviation of the superior visual hemifield was worse than that of the inferior visual hemifield (-12.9±8.1 dB VS -10.4±8.1 dB, p = 0.04). This means that PCT is thinner in areas of progressed glaucoma. Hirooka et al., based on another study [23], reported a relationship between choroidal thickness and glaucoma severity (mean deviation slope) especially in the nasal region 3 mm from the fovea, which is close to the peripapillary choroid, which fact might affect the ONH blood supply. By contrast, in our study, global and localized PCT did not show any correlation with glaucoma severity in the OAG patients. Unlike the present study, neither of the studies by Hirooka et al. employed a localized glaucoma severity parameter such as RNFLT, and the PCT measurement point was different. Confirmation of whether PCT thinning is a localized change corresponding to RNFL defect or a global change regardless of RNFL defect will have to await further study.

Most OCT studies have found that there is no difference in PCT.[11, 14–18] In their multivariate analyses, PCT was correlated with age, AXL, SE but not with glaucoma presence or severity. There are some differences between these studies and the present one. First, the subject groups were different. Among the earlier studies, the subjects of three [15–17] were high tension glaucoma (HTG) patients, of two,[11, 14] OAG patients, and of one,[18] NTG patients. The two OAG studies (conducted in the USA) did not comment on the subject-group composition; it was possible, therefore, that they contained more POAG than NTG patients. Second, the methods of PCT measurement differed. Zhang et al.[11] measured PCT as the mean choroidal thickness of 1 mm x 1 mm squares in the region of the optic disc using SS-OCT. Li et al.[15], Ehrlich et al.[17] and Suh et al.[18] measured PCT as the mean thickness obtained from a 360°, 3.4 mm diameter peripapillary scan using Heidelberg Spectralis EDI-OCT. Maul et al.[14] measured PCT by 12° diameter circular scanning centered on the optic disc using Heidelberg Spectralis EDI-OCT. Hosseini et al.[16] measured PCT at one distal peripapillay point approximately 1 mm from the disc border using Cirrus OCT. As for the third difference between the relevant earlier studies and the present investigation, the latter multivariate analysis showed global PCT to have a significant negative association with disc area in OAG patients. This result suggests that PCT is thinner when measured closer to the disc area. But disc area in the normal controls did not show any association with PCT, and neither was there any significant disc-area difference between the normal controls and the OAG patients (1.90 ± 0.46 VS 1.95 ± 0.39, p = 0.445). On this basis, we supposed that choroidal thinning adjacent to the ONH in OAG patients is due to vascular insufficiency around the ONH. For confirmation of whether PCT is thin or not in NTG compared with normal controls, further studies focusing on PCT measurement adjacent to the ONH, as measured from the end of the Bruch’s membrane of the ONH, will be needed.

Regarding the MCT, there were significant global differences between the two groups in each of the six sectors of the macula. But these did not remain after adjusting for age, AXL, SE. Global, localized MCT in OAG patients were correlated with age, AXL but not with glaucoma severity. These results are consistent with those of the relevant previous studies. The MCT was shown to have a strong relationship with AXL in those six sectors; however, in the advanced stage of glaucoma, it decreased more than in the early or moderate stage, though without statistical significance. Our result differs from those of previous OCT studies. Park et al. and Mwanza et al. reported thicker MCT of OAG patients than of normal controls, though without statistical significance.[13, 21] The increase tended to be greater, moreover, with greater glaucoma severity. They hypothesized that increased MCT was caused by a compensatory response of the choriocapillaries in maintaining blood flow to the macula. This theory, however, does not explain Yin et al.’s reported choroidal thickness decrease, which was observed with reduced choroidal vessel diameter and choroidal filling delay.[8]

Our study has several merits. First, segmentation and measurement of choroidal thickness was performed automatically, using software. Second, to our best knowledge, it is the first study to analyze the relationship between choroidal thickness and various factors in all of the 12 clock-hour sectors of the peripapillary circle and in the six sectors of the macula. Our study also has several limitations. First, given the cross-sectional design, it was difficult to determine whether changes of choroidal thickness preceded glaucomatous damage or followed it. And it was not clear whether the change of choroidal thickness was reversible or irreversible. So, uncovering the exact relationship between choroidal thickness and glaucoma progression will require a prospective study involving multiple choroidal thickness measurements.

In conclusion, in open-angle glaucoma patients, peripapillary choroidal thickness was decreased but not influenced by glaucoma severity. Macular choroidal thickness was not significantly decreased. And choroidal thickness did not show any global or localized relationship with glaucoma severity.

## Supporting Information

S1 Table Choroidal thickness dataset. (XLSX)
